# Giant cell myocarditis in the CMR era

**DOI:** 10.1186/1532-429X-14-S1-M2

**Published:** 2012-02-01

**Authors:** Tora Leong, Joyce Wong, Alexandra Rice, Mamdouh Zidan, Andrew Hamilton, Ben Ariff, Ruth Chester, Shelley L Rahman Haley, Andrew Kelion, Margaret M Burke, Andrew G Mitchell, Nicholas Banner, Tarun K Mittal

**Affiliations:** 1 Harefield Hospital, Royal Brompton & Harefield NHS Foundation Trust, Middlesex, UK

## Summary

Our series of 5 cases of histologically-proven Giant cell myocarditis with concurrent CMR shows a pattern of late gadolinium enhancement which tends to be widespread involving all layers of the myocardium.

## Background

Giant cell myocarditis (GCM) is a rare condition with paucity of data, particularly on diagnosis, prognosis and morphological correlation. We sought to review cases of histologically-proven GCM to examine their presentation and investigations, and in particular, the potential role of cardiac magnetic resonance (CMR) imaging in the diagnosis.

## Methods

Cases of histologically-proven GCM presenting to our institution, a national transplant center, who had concurrent CMR imaging were identified. CMR findings were evaluated from the initial and follow-up scans for ventricular volumes, function, and late gadolinium enhancement (LGE) on an advanced post-processing workstation.

## Results

5 patients with histologically-proven GCM had CMR performed with 2 having repeat CMR. 4 out of the 5 patients were in cardiogenic shock at the time of biopsy (Patients No: 1, 2, 4 and 5). Most patients were initially too unwell for CMR. For the 5 patients, their times from presentation at our institution to time of endomyocardial biopsy (and initiation of treatment soon after) were 0, 3, 11, 0 and 8 days respectively while their times from presentation to first CMR were 12, 31, 9, 56 and 545 days respectively. Table 1 summarizes the CMR findings of these 5 patients and their follow-up. They had moderate to severe reduction in left ventricular (LV) systolic function largely due to increase in end-systolic volumes (ESV) with the end-diastolic dimensions remaining within normal limits. All patients had LGE affecting the myocardium of the LV with multi-focal involvement of all layers of myocardium with no segmental predisposition. 3 patients also had LGE in the right ventricular (RV) myocardium. In the 2 patients who had follow-up CMR, there was deterioration in LV EF due to increasing LV ESV, as well as increasing right ventricular (RV) volumes in the follow-up CMR. These 2 patients received steroids and their immunosuppression regime were azathioprine and ciclosprin (Patient No: 3), and rATG (Patient No: 4). For one of the patients, follow-up biopsy did not show active GCM, raising the possibility of deterioration in LV EF from adverse remodeling.

**Table 1:** 

CMR Findings (Median, IQR) LV EDV (ml) LV ESV (ml) LV EF (%) RV EDV (ml) RV ESV (ml) RV EF (%)	158 (133, 175) 98 (75, 108) 39 (39, 53) 140 (123, 145) 63 (61, 75) 53 (49, 58)
LGE Patterns: Patient No: 1 Patient No: 2 Patient No: 3 Patient No: 4 Patient No: 5	Multifocal mid-wall LGE. Focal sub-endocardial LGE at LV apex and mid-ventricle. Multifocal mid-wall LGE. Thinning of inferior wall at mid-ventricular level. Widespread mid-wall and sub-epicardial LGE with microaneurysms and RV involvement. Widespread mid-wall LGE and extensive sub-epicardial inferior wall LGE. RV involvement. Near global sub-endocardial LGE. RV involvement.

CMR Interval changes: Patient No: 3 LV EDV change LV ESV change LV EF change RV EDV change RV ESV change RV EF change Patient No: 4 LV EDV change LV ESV change LV EF change RV EDV change RV ESV change RV EF change	+5 ml +15 ml -22% +13 ml +24 ml -10% +43 ml +56 ml -35% +53 ml +19 ml +2%

## Conclusions

In the largest available CMR series of histologically-proven GCM, LGE on CMR imaging tends to be widespread involving all layers of the myocardium as opposed to the typical patterns of ‘classical’ myocarditis. This was representative of extensive inflammation and fibrosis which may reflect the high mortality associated with GCM.

## Funding

None.

**Figure 1:**
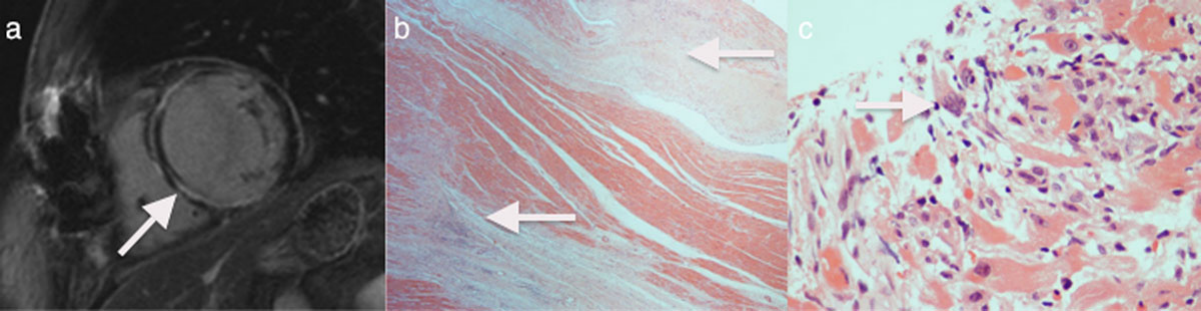
shows (a) the widespread mid-wall LGE in the left ventricle on CMR, (b) lateral wall fibrosis on the explant specimen (H&E, x20), (c) Giant cell myocarditis in pre-transplant diagnostic endomyocardial biopsy (H&E, x200) of Patient No: 4 who had CMR and who subsequently underwent cardiac transplantation.

